# Genetic Polymorphisms in *IGF-I* and *IGFBP-3* Are Associated with Prostate Cancer in the Chinese Population

**DOI:** 10.1371/journal.pone.0085609

**Published:** 2014-02-21

**Authors:** Jian Qian, Hai Zhou, Jiawei Chen, Qi Ding, Qiang Cao, Chao Qin, Pengfei Shao, Pu Li, Hongzhou Cai, Xiaoxin Meng, Xiaobing Ju, Meilin Wang, Zhengdong Zhang, Jie Li, Lixin Hua, Changjun Yin

**Affiliations:** 1 State Key Laboratory of Reproductive Medicine, Department of Urology, The First Affiliated Hospital of Nanjing Medical University, Nanjing, China; 2 Department of Pediatric Surgery, Qiluhospital Shandong University, Jinan, China; 3 Department of Urology, The Changshu Hospital Affiliated to Suzhou University, Changshu, China; 4 Department of Molecular and Genetic Toxicology, School of Public Health, Nanjing Medical University, Nanjing, China; Louisiana State University Health Sciences center, United States of America

## Abstract

*Insulin-like growth factor-I* (*IGF-I*) and *IGF binding protein-3* (*IGFBP-3*) are members of the insulin-like growth factor (IGF) family that play important roles in carcinogenesis. We hypothesized that the functional polymorphisms in *IGF-I* and *IGFBP-3* may be associated with the risk of prostate cancer (PCa) in the Chinese population. This hospital-based case-control study included 664 PCa patients and 702 cancer-free controls. Nine SNPs in *IGF-I* and *IGFBP-3* were genotyped using the TaqMan assay. The genetic associations between the pathogenesis and progression of PCa were assessed by logistic regression. We found that the genotype and allele frequency distribution of rs6218, rs35767 and rs5742612 were significantly different when comparing PCa cases to controls (*P*  = 0.005, 0.005 and 0.020, respectively). In the combined analysis, individuals with 2–6 risk alleles had an elevated risk of PCa compared to those with 0–1 risk alleles. We also found that the association between the combined risk alleles and the risk of PCa appeared stronger in the following subgroups: individuals older than 71 years of age (OR  = 1.41, 95%CI  = 1.05–1.91, *P*  = 0.020), nonsmokers (OR  = 1.68, 95%CI  = 1.21–2.32, *P*  = 0.002), nondrinkers (OR  = 1.32, 95%CI  = 1.02–1.61, *P*  = 0.002), and those with a negative family history of PCa (OR  = 1.28, 95%CI  = 1.02–1.71, *P*  = 0.022). Our results indicate that the three SNPs (rs6218, rs35767 and rs5742612) and the joint genotypes with 2–6 risk alleles, may contribute to the susceptibility to PCa, but not the progression, in the Chinese population.

## Introduction

Prostate cancer (PCa) is the most commonly diagnosed malignant tumor and the second leading cause of cancer mortality in Western men. In 2012, 28170 people died of this malignancy and an estimated 241740 new cases of PCa were expected to be diagnosed in the U.S. [1]. Epidemiological studies have established that PCa morbidity in Asians is much lower [2]. However, in China, as the westernized lifestyle becomes more common, the occurrence of PCa has increased appreciably in recent years [3]. Age, race/ethnicity, genetic background, environmental factors and sex steroid hormone levels are thought to be associated with risk of PCa [4]. Although many people are exposed to these risk factors, only a few individuals develop PCa in their lifetimes, suggesting that genetic variation may contribute to prostate carcinogenesis [5]. Improving results with the candidate gene approach have led to its growing acceptance as a potentially useful method for investigating genetic risk factors for PCa among Chinese.

Insulin-like growth factors (IGFs) are a large family of insulin-related peptides that include *IGF-I* and *IGF-II* as well as their cell surface receptors (*IGF-IR* and *IGF-IIR)*, insulin-like growth factors binding proteins (*IGFBP-1-6*), IGFBP proteases and several other IGFBP-interacting molecules [6], which all regulate cell proliferation, differentiation and apoptosis [7]. The IGFs are bound to several proteins that are involved in different pathways that control cell proliferation and survival including Ras/Raf/mitogenactivated protein kinase (MAPK) [8], phosphatidylinositol 3-kinase (PI3K)/Akt [9] and nuclear factor-κB (NF-κB) [10]. *IGF-I* is located on chromosome 12 and is a 70 amino acid peptide. *IGFBP-3* is located on chromosome 7 and is a 264 amino acid peptide. Both of *IGF-1* and *IGFBP-3* are primarily produced by the liver [11]. The majority of circulating *IGF-I* binds to the main IGF binding protein (*IGFBP-3*); at the same time the *IGFBP-3* regulates the biological activity of *IGF-I*.

Studies have shown that *IGF-I* plays an important role in mitogenesis and antiapoptosis [7], whereas *IGFBP-3* may be antiproliferative and proapoptotic through growth inhibition [12]. Epidemiological studies have confirmed that genetic variations of *IGF-I* and *IGFBP-3* are associated with an increased risk of common cancers, including PCa, colorectal cancer, lung cancer and breast cancer [13], [14], [15], [16].

Given the important role of *IGF-I* and *IGFBP-3* in tumors, we hypothesized that genetic variants of these two genes could have an effect on the risk of PCa. In the present study, five single nucleotide polymorphisms (SNPs) in *IGF-I* (rs6214, rs6218, rs35767, rs5742612, rs5742714) and four SNPs in *IGFBP-3* (rs2132572, rs2854744, rs2854746, rs9282734) were selected and their association with PCa risk in the Chinese population was evaluated.

## Materials and Methods

### Study Population

Between December 2003 and March 2010, 664 untreated PCa patients and 702 control subjects were recruited from the First Affiliated Hospital of Nanjing Medical University, Nanjing, China. All patients were diagnosed via histopathology; control subjects were recruited from among those seeking routine outpatient care and were screened via digital rectal examination (DRE) and presumed to be cancer-free. All subjects were unrelated, ethnic, southern Han Chinese. Each participant was interviewed, in person, by trained interviewers. Detailed information and epidemiological risk factors were collected including age, tobacco use, alcohol use, and family history of cancer. In the present study, individuals who had smoked daily for more than one year were defined as smokers; all others were defined as nonsmokers. Cumulative smoking dose is indicated by Pack-years of smoking (cigarettes per day/20) × years smoked. Individuals who had used alcohol at least three times per week for more than six months were defined as drinkers and the others were defined as non-drinkers. Family history of cancer was defined as any cancer in first-degree relatives (parents, siblings, or children). Disease stage was determined by pathologic findings, pelvic computed tomography, magnetic resonance imaging and radio-nucleotide bone scans. The tumor stage was determined using the international tumor-node-metastasis (TNM) classification and graded according to WHO guidelines. The disease stage was divided into localized and advanced cancer based on the TNM classification system promulgated by the American Joint Committee on Cancer. Localized PCa are those that can be detected clinically or felt (palpated) upon examination, but that have not spread outside the prostate (T_1–2_N_0_M_0_). Advanced PCas are those that have spread outside the prostate (T_3–4_N_x_M_x_, T_x_N_1_M_x_ or T_x_N_x_M_1_). Serum PSA values were estimated by pathologists working at the hospital and divided into two groups PSA >20 ng/ml and PSA ≤20 ng/ml, based on the EAU Guidelines on PCa and on D’Amico’s Risk-Based management of PCa. Each subject donated 5 ml of blood for genomic DNA extraction after having given their written informed consent. The institutional review board of Nanjing Medical University approved the research protocol.

### SNP Selection

Based on HapMap data (http://hapmap.ncbi.nlm.nih.gov/) and PubMed data (http://www.ncbi.nlm.nih.gov/projects/SNP/), we selected 4 SNPs in *IGF-I* (rs6214, rs6217, rs6218, rs35767) and 2 SNPs in *IGFBP-3* (rs2132572, rs9282734). Minor allele frequency (MAF) of all of these genes is more than 5% in the Han Chinese population. Considering a complete linkage disequilibrium (r^2^ = 1) with rs6217, only rs6218 was selected for genotyping. Finally we included one SNP in *IGF-I* (rs5742714) and two SNPs in *IGFBP-3* (rs2854744, rs2854746), which were recently found to be significantly associated with some malignancies in the Chinese population [17], [18], [19].

### Genotyping

Genomic DNA was extracted from anti-coagulated peripheral blood leukocytes by proteinase K digestion and phenol/chloroform extraction. Genotyping was performed with the TaqMan SNP Genotyping Assay. The PCR reactions were carried out in a total volume of 5 µL containing TaqMan Universal Master Mix, 80X SNP Genotyping AssayMix, Dnase-free water and 10-ng genomic DNA. The PCR conditions were 2 min at 50°C, 10 min at 95°C, followed by 40 cycles at 95°C for 15 sec and 60°C for 1 min. The 384-well ABI 7900HT Real Time PCR System (Applied Biosystems, Foster City, CA, USA) was used for the genotyping assay, according to the manufacturer’s instructions and the Sequence Detection Systems software (SDS 2.3; Applied Biosystems) was used to automatically collect and analyze the data and to generate the genotype calls. Four negative controls were included in each plate to ensure accuracy of the genotyping. Two people performed genotyping independently, in a blinded manner, to ensure quality control. Approximately 5% of the samples were randomly selected for repeated genotyping and the results were 100% concordant.

### Statistical Analysis

Pearson’s chi-square (χ^2^) test was used to analyze differences in frequency distributions of demographic variables, pack-years of smoking, smoking status, alcohol use, family history of cancer and genotype frequencies between PCa cases and controls. Using an unconditional logistic regression, we estimated the association between the polymorphisms and risk of PCa by odds ratios (ORs) and their 95% confidence intervals (CIs). All ORs were adjusted for age, cigarette smoking, drinking status and family history. All statistical analyses were two-sided and performed with Statistics Analysis System software (Version 9.1.3; SAS Institute, Inc., Cary, NC, USA) and *P*<0.05 was considered statistically significant.

## Results

### Characteristics of the Study Population

The demographic characteristics and clinical information of PCa cases and controls are outlined in Table 1. There were no significant differences between the PCa cases and controls in the terms of age (*P*  = 0.76). However, the frequency of smokers and drinkers was higher in the PCa case group than in the control group (58.0% versus 50.4%, *P*<0.01; 29.7% versus 24.5%, *P*  = 0.03, respectively). Moreover, there were a significantly higher proportion of PCa cases with a positive family history of cancer when compared with controls (*P*<0.01). Among PCa cases, 389 (58.6%) patients had a PSA level <20 ng/ml. The number and percent of subjects showing a Gleason score <7,  = 7 and >7 was 225 (33.9%), 220 (33.1%) and 219 (33.0%), respectively. Furthermore, 393 (59.2%) patients were in the localized stage and 271 (40.8%) patients were in the advanced stage.

**Table 1:** Distribution of selected characteristics among the PCa cases and control subjects.

Characteristic	Cases (n = 664)		Controls (n = 702)		*P* *
	n	%	n	%	
Age (years)(Mean ± SD)	71.5±8.0		71.3±7.4		0.762
≤71	306	46.1	346	49.3	0.216
>71	358	53.9	356	50.7	
Smoking status					0.005
Nonsmoker	279	42.0	348	49.6	
Smoker	385	58.0	354	50.4	
Pack-years of smoking					<0.001
0	279	42.0	348	49.5	
0–22.5	165	24.9	174	29.8	
>22.5	220	33.1	181	20.7	
Drinking status					0.031
No	467	70.3	530	75.5	
Yes	197	29.7	172	24.5	
Family history of cancer					<0.001
No	538	81.0	647	92.2	
Yes	126	19.0	55	7.8	
Clinical stage					
Localized	393	59.2			
Advanced	271	40.8			
Gleason score					
<7	225	33.9			
= 7	220	33.1			
>7	219	33.0			
PSA(ng/ml)					
≤20	389	58.6			
>20	275	41.4			

*T-test for age distributions between the cases and controls; two-sided χ^2^ test for others selected variables between the cases and controls.

### Distribution of the IGF-I and IGFBP-3 Genotype between the Cases and Controls

Genotype and allele frequencies of the nine polymorphisms among the PCa patients and control subjects, and their associations with risk of PCa, are shown in Table 2. The observed genotype frequencies of the polymorphisms in the control group were consistent with Hardy-Weinberg equilibrium (HWE) (*P*>0.05). As shown in Table 2, we observed significant differences in the distribution of the genotype and allele frequencies of rs6218, rs35767 and rs5742612 between the PCa patients and control subjects (*P*<0.05). For the rs6218 polymorphism, the frequencies of the TT, TC and CC genotypes were 50.1%, 43.7% and 6.2% among PCa cases and 58.3%, 35.0% and 6.7% among controls, respectively (*P*<0.01). The frequencies of the CC, CT, and TT genotypes for rs35767 were 36.5%, 48.6% and 14.9% among PCa cases and 43.3%, 46.6% and 10.1% among controls, respectively (*P*<0.01). Similarly, the frequencies of the TT, TC and CC genotypes for rs5742612 were 44.1%, 44.0% and 11.9% among PCa cases and 51.4%, 39.3% and 9.3%, among controls, respectively (*P*  = 0.02). Based on logistic regression analysis, when using the rs6218 TT genotype as the reference, the TC/CC genotype of the SNP rs6218 was associated with a significantly increased risk of PCa compared with the TT genotype (adjusted OR  =  1.37, 95% CI  = 1.10–1.70). Similarly, a significantly increased risk of PCa was found in the combined genotype rs35767 CT/TT compared with the CC genotype (adjusted OR  = 1.35, 95% CI  = 1.08–1.69). For the SNP rs5742612, the TC (adjusted OR  = 1.30, 95% CI  = 1.03–1.64), CC (adjusted OR  = 1.44, 95% CI  = 0.99–2.09) and TC/CC (adjusted OR  = 1.33, 95% CI  = 1.07–1.66) genotypes were associated with a statistically elevated risk of PCa when compared with the TT genotype.

**Table 2:** SNPs in the IGF-I and IGFBP-3 associated with the prostate cancer risk.

Polymorphisms	Cases (n = 664)		Controls (n = 702)		P *	Adjusted OR (95% CI)†
	n	%	n	%		
IGF-Irs6214						
GG	178	26.8	210	29.9	0.353	1.00(reference)
GA	322	48.5	336	47.9		1.13(0.87–1.47)
AA	164	24.7	156	22.2		1.25(0.92–1.70)
IGF-Irs6218						
TT	333	50.1	409	58.3	0.005	1.00(reference)
TC	290	43.7	246	35.0	0.001	1.42(1.13–1.79)
CC	41	6.2	47	6.7	0.760	1.05(0.67–1.66)
TC/CC	331	49.9	293	41.7	0.003	1.37(1.10–1.70)
T	956	72.0	1064	75.8	0.023	1.00(reference)
C	372	28.0	340	24.2		1.28(1.03–1.61)
IGF-Irs35767						
CC	242	36.5	304	43.3	0.005	1.00(reference)
CT	323	48.6	327	46.6	0.063	1.27(1.00–1.60)
TT	99	14.9	71	10.1	0.002	1.71(1.20–2.44)
CT/TT	422	63.5	398	56.7	0.010	1.35(1.08–1.69)
C	807	60.7	935	66.6	0.002	1.00(reference)
T	521	39.3	469	33.4		1.33(1.04–1.64)
IGF-Irs5742612						
TT	293	44.1	361	51.4	0.020	1.00(reference)
TC	292	44.0	276	39.3	0.021	1.30(1.03–1.64)
CC	79	11.9	65	9.3	0.029	1.44(0.99–2.09)
TC/CC	371	55.9	341	48.6	0.007	1.33(1.07–1.66)
T	878	66.1	998	71.1	0.005	1.00(reference)
C	450	33.9	406	28.9		1.34(1.09–1.61)
IGF-Irs5742714						
GG	448	67.5	492	70.1	0.367	1.00(reference)
GC	195	29.4	195	27.8	0.437	1.08(0.85–1.37)
CC	21	3.1	15	2.1	0.208	1.52(0.76–3.02)
IGFBP-3 rs2132572						
GG	422	63.6	447	63.7	0.820	1.00(reference)
GA	210	31.6	226	32.2	0.893	0.97(0.77–1.23)
AA	32	4.8	29	4.1	0.556	1.17(0.68–2.00)
IGFBP-3 rs2854744						
CC	408	61.4	424	60.4	0.904	1.00(reference)
CA	225	33.9	246	35.0	0.660	0.95(0.76–1.20)
AA	31	4.7	32	4.6	0.980	1.05(0.62–1.78)
IGFBP-3 rs2854746						
CC	398	59.9	420	59.8	0.999	1.00(reference)
CG	228	34.4	242	34.5	0.960	0.99(0.79–1.25)
GG	38	5.7	40	5.7	0.992	0.99(0.62–1.61)
IGFBP-3 rs282734						
AA	598	90.0	636	90.6	0.724	1.00(reference)
AC	61	9.2	63	9.0	0.876	1.03(0.71–1.50)
CC	5	0.8	3	0.4	0.428	1.71(0.40–7.36)

*Two-sided χ^2^ test for either genotype distributions or allele frequencies between the cases and controls.

† Adjusted for age, smoking status, drinking status and family history of cancer in logistic regression model; 95% CI: 95% confidence interval.

### Combined Analysis between the Three Polymorphisms and PCa Susceptibility

Because all three SNPs (rs6218, rs35767 and rs5742612) appeared to be associated with an increased risk of PCa, we combined them, based on the number of the risk alleles, and evaluated the potential interactions of the polymorphisms on the risk of PCa. As listed in Table 3, statistical significance was observed in the combined analysis of risk alleles (*P*<0.01). Furthermore, we classified the risk alleles into two groups according to the number of risk alleles. We found that the risk of PCa was significantly increased in subjects that carried 2–6 risk alleles compared to those carrying 0–1 risk allele(s) (OR  = 1.30, 95%CI  = 1.05–1.62, *P*  = 0.01).

**Table 3:** Stratification analyses between *IGF-I* and *IGFBP-3* genotypes and risk of PCa in cases and controls.

	Cases (n = 664)		Controls (n = 702)		*P* *	Adjusted OR (95% CI)†
	n	%	n	%		
Number ofrisk alleles‡						
0	213	32.3	266	37.9	**0.004**	1.00(reference)
1	74	11.1	85	22.1	0.649	1.13(0.78–1.63)
2	55	8.3	86	12.3	0.250	0.79(0.53–1.17)
3	221	33.3	185	26.4	**0.003**	1.51(1.15–1.99)
4	42	6.3	28	4.0	**0.015**	1.74(1.03–2.95)
5	26	3.9	21	3.0	0.154	1.57(0.85–1.92)
6	33	5.0	31	4.4	0.284	1.30(0.76–2.33)
Recombinedgroups						
0–1	287	43.2	351	50.0	**0.012**	1.00(reference)
2–6	377	56.7	351	50.0		1.30(1.05–1.62)

*Two-sided χ^2^ test for either genotype distributions or allele frequencies between the cases and controls.

† Adjusted for age, smoking status, drinking status and family history of cancer in logistic regression model; 95% CI: 95% confidence interval.

‡ The 0–6 represents the numbers of risk alleles within the combined genotypes; the risk alleles used for the calculation were the rs6218C, rs35767T and rs5742612C alleles.

### Stratification Analysis between the Combined Genotypes and Risk of PCa

The effect on PCa risk of the combined alleles of the three polymorphisms was then evaluated by age, smoking status, pack-years of smoking, drinking status and family history of cancer. As shown in Table 4 **,** we found that the association appeared stronger in the following subgroups: those who were more than 71 years of age (OR  = 1.41, 95%CI  = 1.05–1.91, *P*  = 0.02), nonsmokers (OR  = 1.68, 95%CI  = 1.21–2.32, *P*<0.01), nondrinkers (OR  = 1.32, 95%CI  = 1.02–1.71, *P*<0.01) and those with a negative for family history of cancer (OR  = 1.28, 95%CI  = 1.02–1.61, *P*  = 0.02). We further investigated the association between the combined risk alleles and clinicopathological characteristics of PCa (Table 5). No statistical evidence was found for any interaction between the combined genotypes and progression of PCa.

**Table 4:** Stratification analysis of the variant numbers of genotypes by selected variables in PCa patients and controls.

Variables	Cases (n = 664)				Controls (n = 702)				*P* *	Adjusted OR (95% CI)†
	Number of risk alleles^‡^				Number of risk alleles^‡^					
	0–1		2–6		0–1		2–6			
	n	%	n	%	n	%	n	%		
Total	287	43.2	377	56.8	351	50	351	50	**0.012**	1.30(1.05–1.62)
Age (years)										
≤71	140	45.8	166	54.2	174	50.3	172	49.7	0.247	1.21(0.88–1.67)
>71	147	41.1	211	58.9	211	49.7	179	50.3	**0.020**	1.41(1.05–1.91)
Smoking status										
Nonsmoker	118	42.3	161	57.7	190	54.6	158	45.4	**0.002**	1.68(1.21–2.32)
Smoker	169	43.9	216	56.1	161	45.5	193	54.5	0.665	1.05(0.78–1.41)
Pack-years of smoking										
0	118	42.3	161	57.7	190	54.6	158	45.4	**0.002**	1.68(1.21–2.32)
0–22.5	73	44.2	92	55.8	99	47.4	110	52.6	0.547	1.15(0.76–1.74)
>22.5	96	43.6	124	56.4	62	42.7	83	57.2	0.869	0.95(0.61–1.46)
Drinking status										
No	203	43.5	264	56.5	268	50.6	262	49.4	**0.025**	1.32(1.02–1.71)
Yes	84	42.6	113	57.4	83	48.3	89	51.7	0.280	1.25(0.82–1.89)
Familyhistory of cancer										
No	231	42.9	307	57.1	321	49.6	326	50.4	**0.022**	1.28(1.02–1.61)
Yes	56	44.4	70	55.6	30	54.6	25	45.4	0.211	1.46(0.76–2.81)

*Two-sided χ^2^ test for number of risk alleles in cases and controls.

† Adjusted for age, pack-years of smoking, drinking status, and family history of cancer in logistic regression model; 95% CI: 95% confidence.

**Table 5:** Association between *IGF-I* and *IGFBP-3* polymorphism and clinicopathologic characteristics of PCa.

Variables	Risk allele				*P*	Adjusted OR (95% CI)*2–6 versus 0–1
	0–1		2–6			
	n	%	n	%		
Clinicalstage						
Localized	170	43.3	223	56.7	0.983	1.00(reference)
Advanced	117	43.2	154	56.8		1.00(0.73–1.37)
Gleasonscore						
<7	101	44.9	124	55.1	0.171	1.00(reference)
7	84	38.2	136	61.8		0.77(0.53–1.14)
>7	102	46.6	117	53.4		1.05(0.87–1.26)
PSA(ng/ml)						
≤20	174	44.7	215	55.3	0.351	1.00(reference)
>20	113	41.1	162	58.9		0.85(0.62–1.16)

*Adjusted for age, smoking status, drinking status, and family history of cancer in logistic regression model; 95% CI: 95% confidence interval; OR: odds ratio.

## Discussion

Substantial epidemiologic and experimental evidence, both *in vivo* and *in vitro,* have implicated the IGF pathway as playing a role in prostate carcinogenesis and progression, including cellular metabolism, differentiation, proliferation, transformation, anti-apoptosis, angiogenesis, bone metastases and androgen-independent progression [7], [20], [21]. *IGF-I* can be synthesized and exported by almost all human cells including the prostate tissue [22]. As a potent mitogen, *IGF-I* exerts the mitogenic action of both normal and cancer cells by elevating DNA synthesis, stimulating the cell cycle progression and inhibiting apoptosis [23]. *IGFBP-3,* the most abundant form of IGFBP, binds >90% of *IGF-I*, thus determining the bioavailability of *IGF-I* [24]. *IGF-I* and *IGFBP-3* polymorphisms have been reported to be associated with the risk of PCa in many populations, including Caucasians, African Americans and Japanese [25]. Furthermore, many studies have demonstrated an increased risk of PCa with higher circulating concentrations of *IGF-I* or lower circulating concentrations of *IGFBP-3* [26], [27]. Therefore, we examined whether *IGF-I* and *IGFBP-3* SNPs would affect the risk of PCa in the Chinese population.

In the present study, we assessed the association between nine *IGF-I* and *IGFBP-3* polymorphisms and PCa susceptibility and progression. We found that three SNPs in *IGF-I* (rs6218, rs35767 and rs5742612) were associated with an elevated risk of PCa and that the increased risk was significant among PCa patients that carried the rs6218 TC/CC, the rs35767 CT/TT or the rs5742612 TC/CC genotype. The presence of these risk alleles posed a considerable threat of PCa development in older subjects (>71 years old), non-smokers, non-drinkers and those without a family history of PCa. However, we did not observe any statistical associations between the remaining SNPs and PCa risk.

Recently, the association between the *IGF-I* and *IGFBP-3* polymorphisms and the risk of various cancers, including PCa, has been clarified by many genetic approaches using single amino acid mutations and by molecular epidemiological studies [17], [18], [28], [29], [30], [31]. The conclusion regarding *IGF-I* SNPs in our study is supported by the results of previous studies. Additionally, our results indicated no discrepancy in the genotype distribution of the *IGFBP-3* polymorphisms when comparing PCa cases to controls. Fredrick et al. reported that the variation of the *IGFBP-3* polymorphism (rs2854744) had no association with the risk of PCa among Caucasians [32]. Similarly, Mattias et al. suggested that rs2854744 was not associated with the incidence of PCa and survival in the Swedish population [33]. These results are in accordance with the conclusion of the present study. To our knowledge, this is the first study to evaluate the genetic association between the *IGF-I* and *IGFBP-3* polymorphisms and the risk of PCa in the Chinese population.

*IGF-I*, produced both by liver and locally on the prostate tissue, has effects on prostate carcinogenesis. Epidemiological studies have suggested that a high level of *IGF-I* may increase the risk of PCa in many races [33], [34]. We noted that *IGF-I* levels have been shown to be significantly different between ethnic groups, and a similar result was found among the Chinese [35]. Although the *IGF-I* level is regulated by many factors, about half of the inter-individual variability in serum *IGF-I* can be genetically determined [36]. Considering the relationship of the three *IGF-I* SNPs and PCa risk, we considered whether these genetic variations could be related to the regulation of *IGF-I*. More evidence and research is needed to clarify the association between the three polymorphisms and *IGF-I* level.

In this study, we observed that the rs6218C allele (1.28-fold), rs35767T allele (1.33-fold) and rs5742612C allele (1.34-fold) were associated with a significantly increased risk for PCa. Our results indicated that the rs6218C allele, rs35767T allele and rs5742612C allele were the risk alleles for PCa in the Chinese population. Analysis of the combined alleles was then done in order to obtain a comprehensive estimation of genetic susceptibility for the candidate genes. We found that individuals with joint genotypes containing 2–6 risk alleles had a distinctly higher risk of developing PCa than those whose joint genotypes contained 0–1 risk allele(s). Our results indicated a stronger effect of the alleles on PCa among individuals who were older than 71. This observation is supported by a retrospective study that found that link DNA damage accumulates with increasing age [37]. In addition, we found that the risk of PCa was more pronounced in nonsmokers and nondrinkers. We know that higher circulating concentrations of *IGF-I* may contribute to a higher risk of PCa and a previous study by Libby M *et al.* reported that circulating *IGF-I* was influenced by lifestyle factors [38]. One explanation for the higher risk of PCa in nonsmokers and nondrinkers may be that environmental effects overwhelm the effects of genetics.

Some limitations of this study should be noted. First, the sample size was moderate, which limited the statistical power of combined analysis and stratification. Second, the lack of detailed information on cancer risk factors such as diet, physical activity and occupational exposure further limited evaluating the associations between environmental factors and PCa risk. Third, our study was a retrospective hospital-based study so the inherent selection bias could not be entirely excluded. In addition, considering the limitations of DRE, we might have had some misclassification of disease among control subjects. However, all genotype frequencies of the nine polymorphisms that occurred in PCa patients and in controls subjects in this study were in agreement with HWE, suggesting that the selection bias was unlikely to be substantial.

In conclusion, the present study provides evidence to elucidate the genetic effects of the *IGF-I* and *IGFBP-3* SNPs on the pathogenesis and progression of PCa. We found three polymorphisms in *IGF-I* (rs6218, rs35767 and rs5742612), and their combined alleles, that might elevate the risk of PCa in the Chinese population. Further epidemiological studies with larger sample size and more environmental and survival factors are needed in order to confirm our findings.
